# Synthetic versus analytic approaches to protein and DNA structure determination

**DOI:** 10.1007/s10539-018-9636-0

**Published:** 2018-07-04

**Authors:** Agnes Bolinska

**Affiliations:** Department of History and Philosophy of Science, University of Cambridge, Free School Lane, Cambridge, CB2 3RH UK

**Keywords:** Heuristics, X-ray diffraction crystallography, Evidence, Protein structure, DNA structure, Alpha helix, Information, Discovery

## Abstract

The structures of protein and DNA were discovered primarily by means of *synthesizing* component-level information about bond types, lengths, and angles, rather than *analyzing* X-ray diffraction photographs of these molecules. In this paper, I consider the synthetic and analytic approaches to exemplify alternative heuristics for approaching mid-twentieth-century macromolecular structure determination. I argue that the former was, all else being equal, likeliest to generate the correct structure in the shortest period of time. I begin by characterizing problem solving in these cases as proceeding via the elimination of candidate structures through the successive application of component-level information and interpretations of X-ray diffraction photographs, each of which serves as a kind of *constraint* on structure. Then, I argue that although each kind of constraint enables the elimination of a considerable proportion of candidate structures, component-level constraints are significantly more likely to do so correctly. Thus, considering them before X-ray diffraction photographs is a better heuristic than one that reverses this order. Because the synthetic approach that resulted in the determination of the protein and DNA structures exemplifies such a heuristic, its use can help account for these discoveries.

## Introduction

Within a few years in the mid-twentieth century, Linus Pauling^1^ determined the structure of protein and Watson and Crick discovered the structure of DNA. In both cases, the eventual discoverers of these structures competed with rival groups in other labs. And in both cases, the discoverers and their rivals approached the problem differently. Pauling, together with collaborator Robert Corey, adopted a bottom-up, compositional approach^2^: he took what he knew about different structural components of protein—bond types,^3^ lengths, and angles between atoms in the polypeptide chain—and used these as puzzle pieces, so to speak, from which the three-dimensional structure could be built up (Judson 1996, p. 62). Inspired by Pauling’s success, Watson and Crick adopted a similar strategy, constructing three-dimensional models from structural features of DNA. Their rivals, Sir Lawrence Bragg, John Kendrew, and Max Perutz in the case of protein and Rosalind Franklin in the case of DNA,^4^ instead adopted a top-down, decompositional approach: rather than attempting to *synthesize* information about what was known about the individual components of the molecules, these groups instead *analyzed* X-ray diffraction photographs of the molecules in question.

One might ask, to what extent was this difference in approach responsible for the eventual successes of the discoverers in each case? Although I will propose an answer to this question, this will be only my secondary aim. My primary aim will be to address a related set of issues. Suppose a beginning graduate student wishing to work on the structure of one of these macromolecules were selecting between labs to join. Which should she choose? Or suppose a granting agency were attempting to determine which research project to fund. Which is most promising? The driving question: given the state of scientific knowledge and technology at the time, which of these approaches—the synthetic one championed by Pauling and Watson and Crick, or the analytic one favoured by Bragg’s group and Franklin—had the greatest likelihood of finding the molecular structure in question in the shortest period of time?

This is a question about *heuristics*, problem-solving guidelines or “rules of thumb.” There is no algorithm for solving complex scientific problems like those of protein and DNA structure. Scientists do not have a set of instructions that, if precisely followed, will lead them to the correct structure. Rather, a degree of trial and error is involved. Scientists follow promising leads and make false starts; they propose and abandon various hypotheses. The process is messy, yet not directionless: it is guided by some overarching principles, even if scientists deviate from them from time to time. So to say, for instance, that one has followed the heuristic of the synthetic approach, one need not have considered *all* component-level considerations before looking at X-ray diffraction photographs. Rather, one follows the heuristic so long as one *in general* considers such constraints first. Thus, Pauling can be understood as having followed the heuristic even if, as we will see in “A (concrete) heuristic: average informativeness of constraints on molecular structure” section, he went back and forth between reasoning from known structural components of protein and X-ray diffraction photographs to some extent.

Wimsatt (2006, pp. 464–65) identifies several important properties of heuristics, four of which will be particularly relevant for what follows. First, although heuristics might increase the likelihood of success, they do not *guarantee* it, in contrast to truth-preserving algorithms, which guarantee the truth of the conclusion given the truth of the premises. This is in part because heuristics impose far fewer restrictions on how one should proceed, and therefore even if one adopts the best possible heuristic, other factors can affect whether or not one succeeds. Second, heuristics are “*cost*-*effective*…in terms of demands on memory, computation, or other limited resources” (Wimsatt 2006, p. 465, emphasis original). Third, applying a heuristic to a particular problem amounts to transforming that problem into a non-equivalent, but related problem. Thus, when a problem is solved using a heuristic, there remains the question of whether it is appropriate to consider the solution to the transformed problem to also be a solution to the original problem. Finally, heuristics are purpose-relative; that is, they are useful for certain aims, but not others.

Which heuristic was best for approaching the problem of determining the structure of a complex macromolecule such as protein or DNA in the mid-twentieth century? In particular, which was likeliest to be the most “cost-effective” in the sense of the second property above? In order to answer these questions, let us begin by characterizing different pieces of empirical evidence and theoretical considerations as *constraints* on molecular structure: in order to be considered acceptable, a structure must accord with or account for each such piece of evidence and theory. We may then characterize the synthetic and analytic approaches as differing with respect to the *order* in which different constraints on structure are taken into account. The synthetic approach can be understood as beginning by taking into account information about bond types, lengths, and angles, considering X-ray diffraction photographs of the molecule in question only afterwards, while the analytic approach can be regarded as reversing this order, deriving what can be known from such X-ray diffraction photographs first, and then comparing that with information about bond types, lengths, and angles.

The order in which different considerations, particularly pieces of empirical evidence, are considered is not usually taken to have any bearing on the outcome of an investigation. Indeed, Bayesians assume that an adequate model of updating beliefs ought to be commutative, i.e. updating on evidence A before evidence B should produce the same degree of confirmation as updating on B before A, and a common criticism of Jeffrey Conditionalization is that it fails to meet this requirement (cf. Domotor 1980; Doring 1999; Field 1978; Skyrms 1986; van Fraassen 1989). But the view that the order in which evidence is consulted should not affect the outcome of an investigation rests on the assumption that the time available for its completion is infinite. In practice, of course, this is not the case.^5^ Research is often driven by competition between groups, and in order to make a discovery *at all*, one must make it *first*, beating one’s rival to it. In actual scientific practice, speed and efficiency matter.

I will argue that the order in which different constraints are taken into account affects how quickly or efficiently a problem is likely to be solved, thereby contributing to the “cost-effectiveness” of a heuristic. My goal is to demonstrate not only *that* this order matters, but also to explain *why* it does. In order to do so, we may understand the problem of molecular structure determination as beginning with a space of candidate structures, each a possible solution to the problem, the size of which is reduced through the consideration of various constraints, thereby transforming the original problem into a related problem, in accordance with the third property of heuristics above.^6^ The consideration of a particular constraint requires its application to the problem, which can involve interpretation: what does it tell us about which solutions are possible? As we will see, such an understanding of the problem enables us to estimate what I will call the *average informativeness* of a constraint, a function of two variables: (1) the extent to which its application reduces the size of the possibility space, leaving fewer structures for further consideration, and (2) how confident scientists could be that they had *correctly* applied the constraint, that is, eliminated only incorrect structures through its application.

Developing an understanding of how to select between heuristics is significant for two reasons. First, scientists often make decisions about how to approach complex problems in the face of various kinds of uncertainties: about the truth, accuracy, or instrumental value of relevant theories and models, about the feasibility and applicability of experimental procedures, and about the reliability of instruments and interpretation of results, not to mention other social and pragmatic factors. These decisions are far from straightforward. Thus, an analysis of how to approach them is valuable in its own right. However, such an analysis can also shed new light on two well-known historical cases, providing at least a tentative answer to the question of how a difference in approach might have contributed to the successes of the discoveries of the protein and DNA structures. I will argue that, to the extent that the problems and approaches to solving them adopted by the historical actors reflect the transformed problems and heuristics that will be the focus of this paper, we ought to accept the answer to the question of which heuristic was best as also explaining—perhaps only partially, and in conjunction with other proposals—the successes of the groups that made the discoveries. And I will further argue that there is, indeed, significant overlap between the idealized problems and heuristics I describe here and the approaches adopted by the different groups in these cases.

The paper proceeds as follows. I begin by introducing the problems of protein and DNA structure as they stood in the mid-twentieth century, describing the ways in which different theoretical and empirical considerations constrained which structures were permissible. I show that the process of structure determination may be understood as proceeding via the successive elimination of candidate structures, with each constraint dictating which structures are to be eliminated (“The transformed problem: reducing the space of structural possibilities by applying constraints on structure” section). Then, I introduce the notion of average informativeness. I argue that the best heuristic is the one that considers the most informative constraint first (“An (abstract) heuristic: weighing confidence in correct constraint application against degree of possibility space reduction” section). I show that, in our case studies, component-level constraints concerning bond types, distances, and angles were more informative than whole-molecule X-ray diffraction photographs, so taking the former into account before the latter was most likely to yield the correct structure first (“A (concrete) heuristic: average informativeness of constraints on molecular structure” section). I discuss the extent to which the transformed problem of molecular structure determination reflects the actual problem (“Does the transformed problem reflect the real problem?” section), and conclude by suggesting that Pauling’s and Watson and Crick’s having followed this heuristic can help account for their discoveries of the protein and DNA structures respectively (“Denouement: explaining Pauling’s and Watson and Crick’s successes” section).

## The transformed problem: reducing the space of structural possibilities by applying constraints on structure

**Fig. 1:**
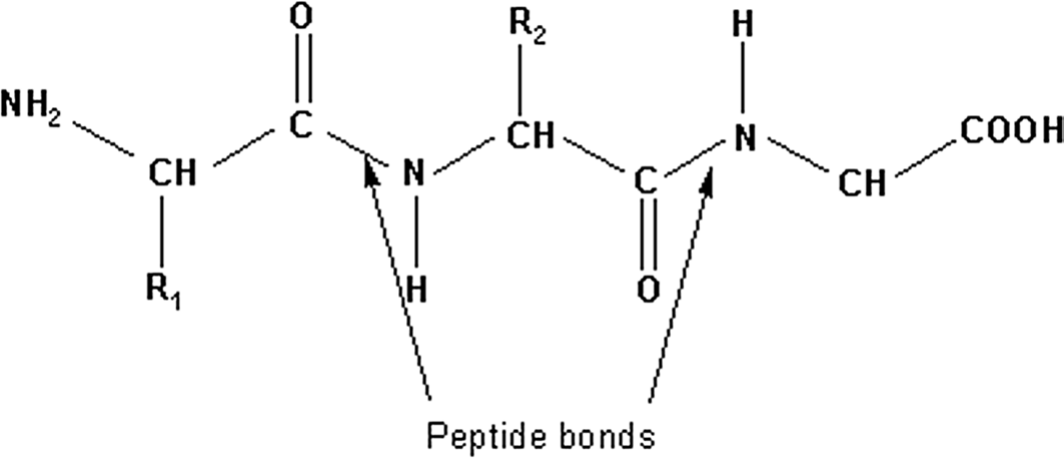
The structure of a polypeptide chain, with the R groups corresponding to different amino acids and peptide bonds indicated. The problem of protein structure was to determine how polypeptide chains folded in three dimensions

**Fig. 2:**
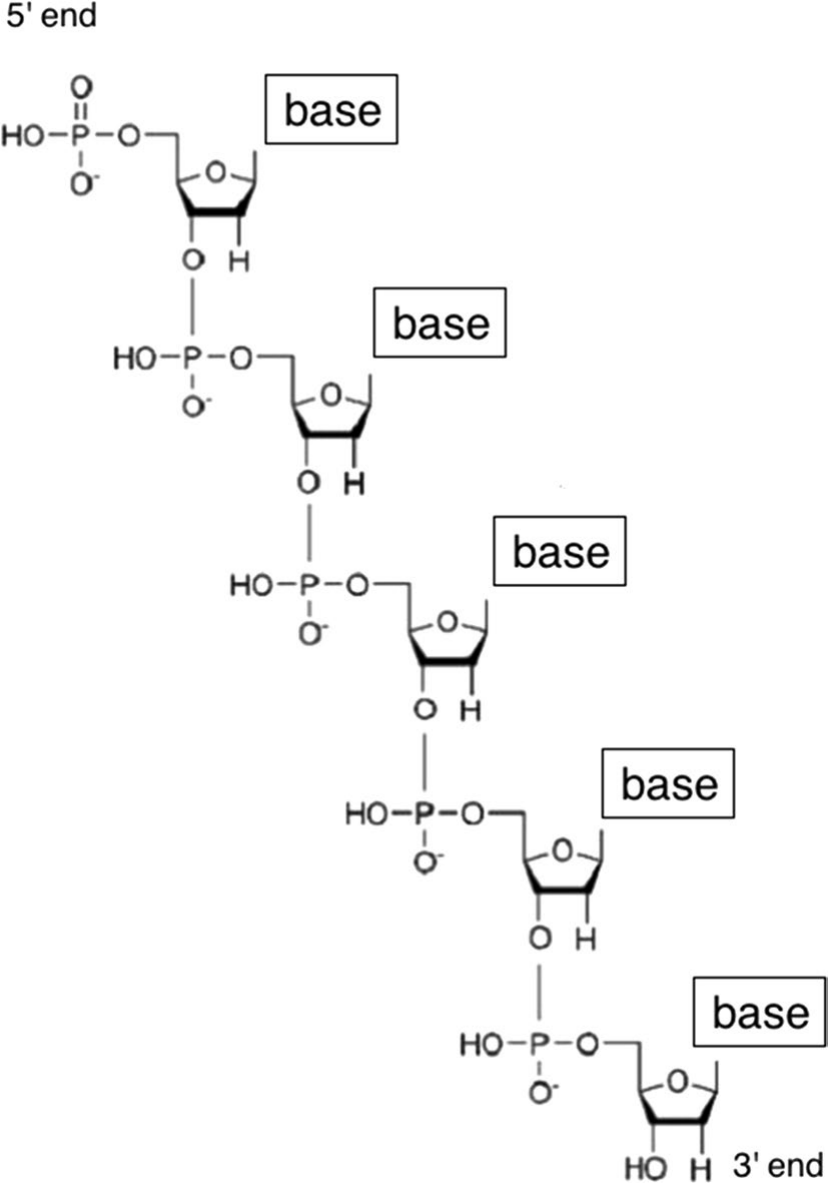
A sugar-phosphate chain with 3′ and 5′ ends labeled

The mid-twentieth-century problems of protein and DNA structure were to determine how one or more chains of molecular subunits folded in three dimensions. In the case of protein, the subunits in question were amino acids, each with the same basic structure but differentiated from one another by unique R groups. Strings of amino acids were known to be connected by peptide bonds into *polypeptide chains* (Fig. 1). In the case of DNA, the subunits were nucleotides, each made up of a phosphate group, the sugar deoxyribose, and one of four bases: adenine, guanine, cytosine, or thymine. The sugar–phosphate chains they formed had directionality in the sense that they had two distinct ends, known as the 3′ and 5′ ends (Fig. 2). The problem was to determine how many such chains DNA contained, how they were oriented with respect to one another, and how the molecule folded in three dimensions.

In each case, the problem may be characterized as follows. One begins with a space of possible structures, defined by what was known about the molecule. In the case of protein, the set of candidate structures was composed of the various ways the polypeptide chain might fold—its possible *conformations*. In the case of DNA, this set was larger, consisting of the various possible permutations of two or three sugar–phosphate chains, arranged in parallel or anti-parallel, with the bases on the inside or outside of the molecule, and again all of the possible ways in which the molecule could fold. The size of this space is initially very large, since bond lengths and angles could be varied continuously and variously combined with one another.^7^ It may be reduced through the consideration of different constraints on structure, bits of information about bond types, lengths, and angles and X-ray diffraction photographs of the molecule.

How such reductions of the possibility space take place depends on the type of constraint under consideration. Let us consider each in turn. To see how information about bond types, lengths, and angles constrains structure, enabling the successive reduction of the size of the possibility space, let us consider a simplified example. Suppose that we are trying to determine the structure of formamide, and we know it to have the following structural formula:
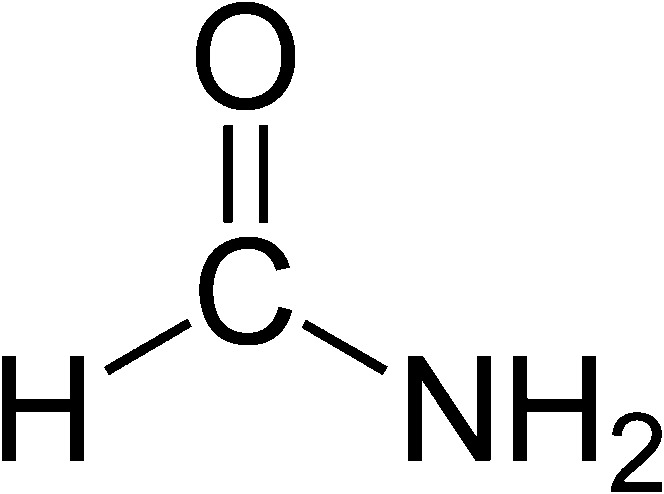


Suppose that we also know the bond angles and distances between the atoms in formamide. We may use these as constraints enabling the successive elimination of candidate structures as follows. Prior to the application of a constraint, very many structures are possible, since each of the bond lengths and angles in the molecule can take on any value. If we apply the information that the C–N bond distance is 1.34 Å, we may eliminate candidate structures with C–N bond distances other than 1.34 Å from further consideration.^8^ If we then consider the fact that the C=O bond distance is 1.24 Å, we may again eliminate further structures from the possibility space, leaving only those with 1.34 Å C–N and 1.24 Å C=O bond distances. We may continue in this fashion, thereby fixing the bond distances in the molecule. Similarly, applying bond angles as constraints enables us to eliminate any structures that do not conform with them. Finally, bond types also constrain structure in the same way.^9^ A molecule may only rotate about a single bond. Moreover, due to a phenomenon known as resonance, some bonds that are depicted in structural formulae as single bonds in fact have partial double-bond character. The peptide bond in formamide—the same bond that joins amino acids together to form a polypeptide chain—is an instance of such a bond: an electron from the C=O bond spends some of its time at the C–N bond. Thus, rotation about the peptide bond is also prohibited. Upon taking each component-level constraint into consideration, successively narrowing down the space of possible structures, we are left with only one, the planar molecular structure of formamide.

Let us now turn to the way in which X-ray diffraction photographs of a molecule constrain the space of possible structures. X-ray diffraction photographs are produced by shining a beam of X-rays through a regular lattice with repeating subcomponents, such as a crystallized long-chain molecule. The electron clouds of the atoms in the molecule scatter X-rays, producing a diffraction pattern on a photographic plate. The locations and intensities of spots on a photograph form a *reciprocal lattice*, which contains information about the amplitude of the X-rays. However, in order to determine atomic positions from these photographs, not only the amplitude but also the phase of the X-rays is required. Thus, the photographs contain only partial information about structure. This is known as the *phase problem*.

**Fig. 3:**
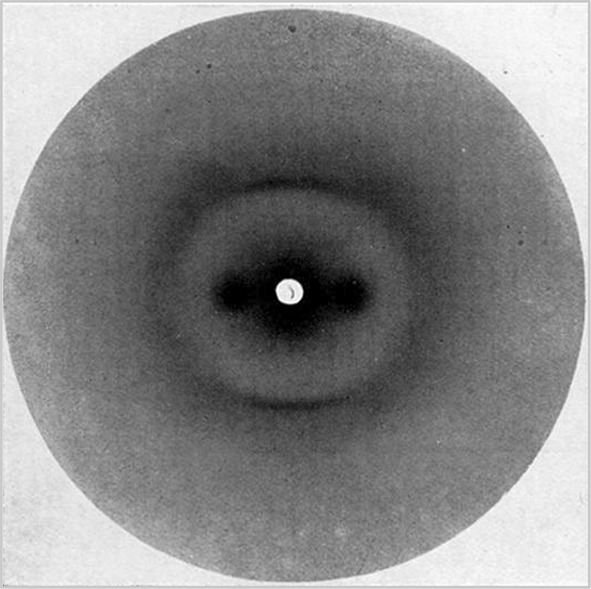
One of Astbury’s X-ray diffraction photographs of keratin. Reproduced from Astbury and Street (1932)

Thus, in order to use X-ray diffraction photographs as constraints on structure, one had to first interpret them to determine what structural information they provided. This should be borne in mind in the following illustrations, and will be important in the discussion in “A (concrete) heuristic: average informativeness of constraints on molecular structure” section. At the University of Leeds, crystallographer William Astbury produced numerous X-ray diffraction photographs that would figure crucially in the determination of the protein and DNA structures. In the 1930s, he showed that there were two forms of keratin, a protein that makes up wool, fingernails, and hair: unstretched (which he named ‘alpha’ keratin) and stretched (‘beta’) keratin. A characteristic spot in Astbury’s photographs was taken to indicate that alpha keratin had a subunit that repeated every 5.1 Å (Fig. 3).^10^ Taking this spot into consideration constrained protein structure in the sense that it enabled one to eliminate any structure lacking such a repeating subunit from the space of possibilities. Similarly, Astbury’s photo of DNA, taken before the war and published in 1947, was taken to show that DNA contained a monotonous repetition of the four nucleotides with 3.4 Å between each, that it had a large structural repeat every 27 Å, and that the sugar component was parallel to the flat bases. Again, this photo would constrain DNA structure by eliminating from the space of possibilities structures that did not contain these features. Finally, as is well known, Franklin also produced a great number of clear diffraction photographs of DNA. Her most significant finding was that DNA could take on one of two discrete forms: a crystalline ‘A’ form, or a wet ‘B’ form. Franklin discovered that the DNA sample in Astbury’s photo was a mixture of the two forms. By the spring of 1952, she had a number of very clear images of both the A and B forms (Fig. 5). The distinct ‘X’ pattern in the photo of the B form suggested a helical conformation for DNA, enabling the elimination of non-helical structures from the space of possibilities.

**Fig. 4:**
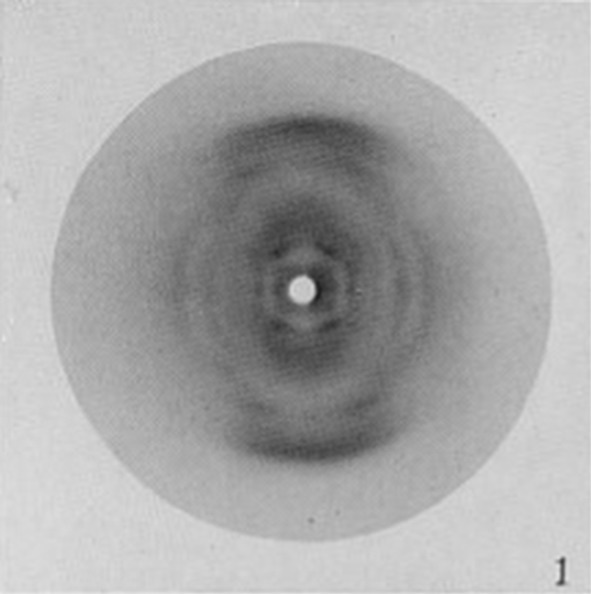
Astbury’s X-ray diffraction photograph of DNA. Reproduced from Astbury (1947)

**Fig. 5:**
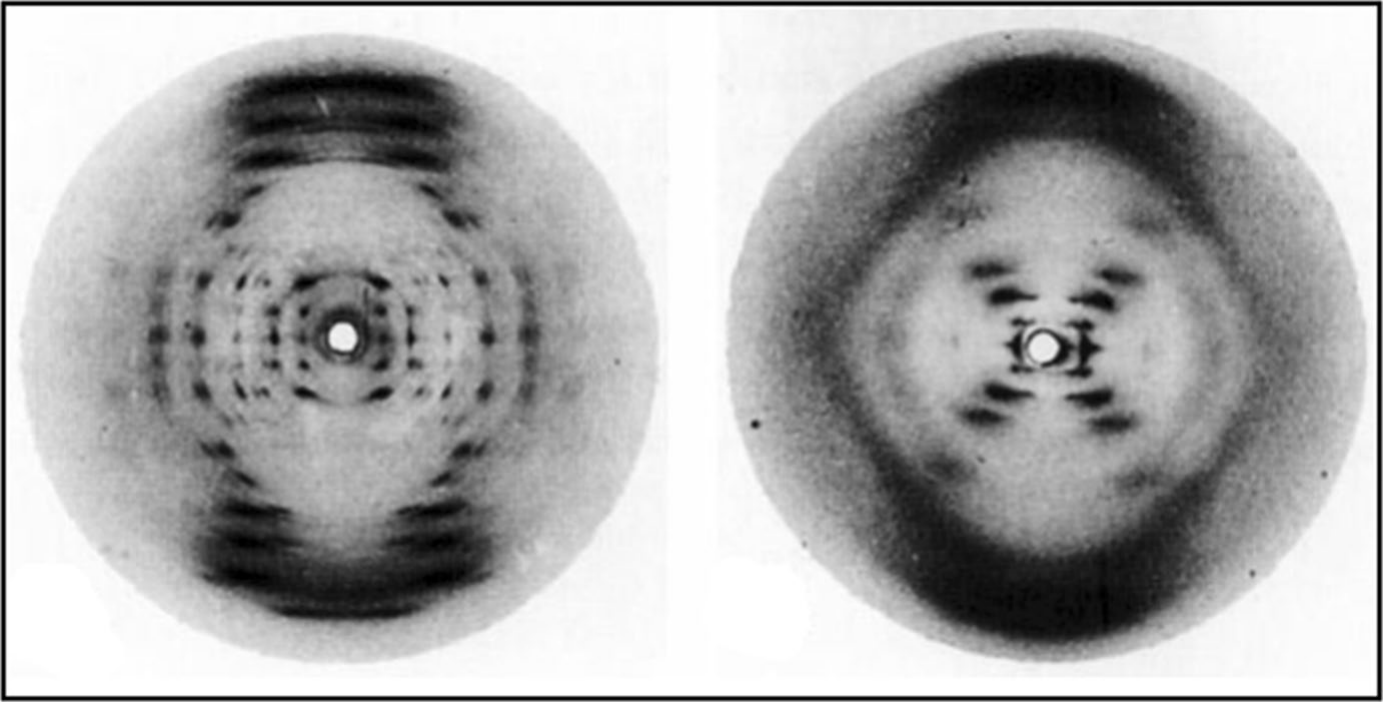
Examples of Franklin’s diffraction photos of the A form (left) and the B form (right). Reproduced from Franklin and Gosling (1953)

We have seen how both component-level information about bond types, lengths, and angles and whole-molecule X-ray diffraction photographs may constrain molecular structure by enabling the successive elimination of candidate structures incompatible with them from the possibility space. If one’s aim is to determine the correct structure of a molecule as quickly as possible, one ought to reduce the size of the possibility space as much as possible with each constraint one considers. Thus, if one had full confidence in one’s interpretation of each constraint—and hence also in the reduction of the possibility space resulting from its consideration—then those constraints that eliminate the greatest number of structural candidates, leaving the fewest remaining for further consideration, ought to be taken into account first.

But in scientific practice, one may never have full confidence in one’s interpretation; at best, one may hold a high degree of belief that it is correct. So a cost-effective heuristic must not only maximize how many structural candidates are eliminated with each constraint; it must also maximize the degree to which scientists can be confident they have eliminated only those structures that are incorrect through its application. For if the application of a constraint mistakenly eliminates the correct structure, removing it from the space of possibilities remaining for further consideration, one will be led astray, and might end up having to backtrack or start the process again. This is precisely what happened to Bragg, Kendrew, and Perutz. In a paper published in the spring of 1950, they considered twenty candidate structures for alpha keratin, and selected from among them the one they thought most likely to be correct on the basis that it that best accounted for Astbury’s 5.1-Å spot (Bragg et al. 1950), eliminating a structure that was fairly similar to the alpha helix, the correct structure eventually discovered by Pauling (Olby 1974, pp. 289–90). Thus, scientists ought to begin by considering those constraints that eliminate the greatest number of candidate structures with the greatest certainty.

But things are not always so simple, since those constraints that eliminate the greatest number of structural candidates may not be those in which scientists have the greatest confidence. It may be the case that a given constraint eliminates many possibilities, but scientists are not very certain that it is correct. Alternatively, they might be reasonably confident in a particular constraint that eliminates very few possibilities. So how ought they determine the order in which to apply different constraints?

## An (abstract) heuristic: weighing confidence in correct constraint application against degree of possibility space reduction

I will argue that scientists must weigh the number of structural possibilities that the application of a given constraint eliminates against how certain they can be that it has eliminated only incorrect ones. I will begin by describing the game Twenty Questions and discussing the order in which questions ought to be asked in this game to maximize one’s chances of winning (“A heuristic for twenty questions” section). Then, I will argue that playing Twenty Questions is importantly analogous to the process of protein and DNA structure determination, so we can apply some of what we know about the former case to the latter (“Twenty questions and molecular structure determination” section).

### A heuristic for Twenty Questions

In the game Twenty Questions, one player brings to mind some entity or being—let us call it the ‘object of inquiry’—and the other tries to guess what it is by asking a series of yes-or-no questions. Since the maximum number of questions permitted is twenty, the goal is to reveal the identity of the object of inquiry by asking as few questions as possible. What is the best question to ask at each stage in Twenty Questions? Suppose we have reached a stage in the game where we know that the object of inquiry is a whole number between 1 and 100. In this case, there are one hundred possible answers left. Consider three candidates for the next question:

Q1. Is the number 1?

Q2. Is it greater than 80?

Q3. Is it even?

Asking Q1 carves out the remaining possibilities into groups of one and ninety-nine. If the answer is ‘Yes’, we are left with only one possibility and the game is over, whereas ‘No’ leaves us with ninety-nine remaining for further consideration. Thus, the probability that only one out of one hundred possibilities will be eliminated is 0.99, whereas the probability that ninety-nine possibilities will be eliminated is 0.01. Asking Q2 divides remaining possibilities into groups of twenty (if the answer is ‘Yes’) and eighty (if the answer is ‘No’); the probabilities that twenty and eighty possibilities will be eliminated are 0.8 and 0.2 respectively. Finally, asking Q3 divides the possibility space in half: fifty possibilities for ‘Yes’, fifty for ‘No’. Thus, the probability that fifty possibilities will be eliminated is 1.

The best heuristic for playing Twenty Questions is to ask questions like Q3, those that divide the possibility space in half, or as close to half as possible. Although we might get lucky in asking Q1 and get the right answer in just one question on some particular play of the game, this would happen rarely. At earlier stages in the game, getting the right answer by asking questions like Q1 would be even more rare: if we had determined only that the object of inquiry was a whole number between one and a thousand, we would have a 0.001% chance of guessing its identity in just one question. This probability would be even lower if we only knew that the object of inquiry was a whole number, or if we did not know that it was a number at all.

Q3 is a better question than Q2 and especially Q1 because it finds the best balance between how many possibilities might be eliminated and how certain one can be that this many possibilities will in fact be eliminated. That is, on average, Q3 eliminates more possibilities than Q2 or Q1. We can be reasonably certain, for instance, that the answer to Q1 will be ‘No’ because the probability that the answer will be ‘No’ is 0.99. Although we might get lucky if the object of inquiry is 1, *on average*, we do not learn much by asking Q1: usually, we just learn that the number is not 1, eliminating only one of one hundred possibilities. So we should ask questions like Q3 first, asking questions like Q1 and Q2 only in later stages of the game.

### Twenty Questions and molecular structure determination

There are several similarities between playing Twenty Questions and determining protein or DNA structure. First, the best heuristic for both is whichever one most reliably finds the shortest route to success: in Twenty Questions, the one that minimizes the number of questions that must be asked to determine the identity of the object of inquiry, and in the process of molecular structure determination, the one that minimizes the number of inferential steps required to find the correct structure. Second, just as different considerations in our case study act as constraints on molecular structure, so too do question–answer pairs in Twenty Questions, suggesting possible identities for the object of inquiry while eliminating others. Finally, both Twenty Questions and molecular structure determination involve some degree of uncertainty at each step: the answer to a question cannot be predicted, and one cannot know whether one’s application of a constraint to eliminate particular candidate structures is correct.

However, while both Twenty Questions and the process of molecular structure determination involve some degree of uncertainty at each step, the nature of this uncertainty is different in each case. In Twenty Questions, a player cannot predict what the answer to a question will be. As we saw in the last section, her uncertainty is precisely quantifiable: the probability of each answer is the proportion of possibilities remaining in the possibility space that are compatible with that answer. In contrast, the uncertainty in the process of molecular structure determination resides in scientists’ not knowing whether a given application of a constraint is correct, and thus not knowing whether the correct structure does, indeed, lie in the space of possible structures remaining for further consideration. In Twenty Questions, a player is uncertain how much the space of possible structures will be reduced when a question is asked; but once she receives the answer, her uncertainty is dissolved, and she is left with a new, definite possibility space that helps her decide which question to ask next. In the case of molecular structure determination, the uncertainty in a scientist’s application of a constraint remains once that application has been made. Moreover, it ‘infects’ the remainder of the process in the sense that if the application is incorrect, subsequent reasoning about the structure is led astray.

Although the kind of uncertainty inherent in applications of constraints in the case of protein structure determination is different from that which exists upon asking a question in Twenty Questions, both kinds of uncertainty have the same effect: they detract from the efficiency of the process at hand. Thus, in both cases, one has an imperative to reduce this uncertainty as much as possible. In fact, this imperative is greater for the process of molecular structure determination, since the kind of uncertainty present there has the potential to lead scientists off course, putting them at risk of having to backtrack and determine where they went wrong. Such a costly error is simply not possible in Twenty Questions. Moreover, the detractions from the efficiency of the process that both contributions make are similar in degree. By asking questions like “Is the number 1?” at each stage, a player is highly unlikely to guess the identity of the object of inquiry in just twenty questions; similarly, by applying constraints in a way that is unlikely to be correct, one probably will not determine the correct structure of protein or DNA in a timely manner.

Despite this difference in kinds of uncertainty, important features of the analogy between Twenty Questions and the process of molecular structure determination are preserved: maximizing the number of possibilities eliminated with each step contributes to the efficiency of the process, as does minimizing the amount of uncertainty present at each juncture. We may capture these features using what I will call *average informativeness*, or simply *informativeness* for short, to determine the order in which to consider different constraints.^11^ The informativeness of constraint is a function of (1) the extent to which its application reduces the size of the possibility space and (2) the extent to which scientists were warranted in believing that the application is correct. Although (1) and (2) cannot be precisely quantified, as I will show in the next section, their relative values can nonetheless be estimated. So just as the best heuristic for Twenty Questions is to ask questions that reduce the space of possible answers with the greatest certainty each time, the best heuristic for determining protein or DNA structure, other things being equal, is to consider the most informative constraint at each stage.^12^

## A (concrete) heuristic: average informativeness of constraints on molecular structure

In the last section, I introduced an abstract heuristic for determining molecular structure: different constraints should be applied in order of decreasing informativeness, where informativeness is a function of the extent to which the application of a constraint reduces the size of the space of possible structures and how confident scientists could be that this application is correct. In this section, I will argue that component-level constraints were, in this sense, more informative than X-ray diffraction photographs of the molecule in the cases of protein and DNA structure determination: although both kinds of constraints enabled the elimination of a considerable proportion of the possibility space, the confidence that scientists were warranted in having in their applications of component-level constraints significantly outweighed the confidence they were warranted in having in their interpretations of X-ray diffraction photographs. Thus, the best (concrete) heuristic for molecular structure determination in these cases was to apply component-level constraints to eliminate possible structures first, only afterwards eliminating further structures using interpretations of X-ray diffraction photographs.

Let us consider each of the factors that contribute to the informativeness of a constraint in turn, beginning with the extent to which its application enables a reduction of the size of the possibility space. To approximate the extent to which the application of a component-level constraint reduces the size of the possibility space, consider again the example of determining the structure of formamide (“The transformed problem: reducing the space of structural possibilities by applying constraints on structure” section). Applying the constraint of the 1.34-Å C–N bond distance eliminated fewer than, say, a tenth of the possibilities. For even once we fix the C–N bond distance, our possibility space still includes structural candidates with any bond angles and distances for all of the other bonds in the molecule. On the other hand, Astbury’s interpretation of his X-ray diffraction photograph of alpha keratin certainly eliminated more than half, possibly more than three-quarters, of remaining possibilities: it reduced the possibility space from including *any* configuration to only those with a repeating subunit every 5.1 Å.

The proportions of eliminated structures I have here indicated are meant only as first-pass, rough-and-ready estimates. My aim is merely to show that although the interpretation of an X-ray diffraction photograph eliminates a greater proportion of the remaining structures than the application of a component-level constraint, the latter is nonetheless able to eliminate a significant number of structural possibilities. This is important. If the number of structures eliminated by the application of a component-level constraint were sufficiently small, it would not matter how confident scientists were in this application; it would be better to begin instead by examining those constraints in which they had less confidence but which permitted the elimination of a greater number of structural candidates. But the disparity between the number of structures eliminated by the application of component-level constraints and by interpretation of X-ray diffraction photographs is not quite so great: component-level constraints and X-ray diffraction photographs both reduce the size of the space of structural candidates remaining for further consideration to a significant degree.

Let us turn now to the second factor contributing to a constraint’s informativeness. How confident could scientists be in their applications of each kind of constraint? That is, how certain could they be that, upon the elimination of possibilities through the application of a particular constraint, the correct structure remained in the set of structural candidates remaining for further consideration? The certainty that one is warranted in having in the application of a constraint is a product of at least two considerations: (1) the number of competing possibility space reductions compatible with that constraint and (2) the reliability of the constraint itself. If there are several ways to reduce the possibility space by applying a particular constraint, there exists the possibility that one chooses among them incorrectly, mistakenly removing the correct structure from further consideration. Similarly, if the constraint itself is unreliable, one may also be led astray by eliminating structural possibilities on its basis. I will show that, in our case studies, component-level constraints fared far better than X-ray diffraction photographs on both these fronts.

Each component-level constraint can generally only be applied in one correct way. Consider again the example of determining the structure of formamide by applying different such constraints in succession. Applying the 1.34-Å C–N bond distance to constrain structural possibilities amounts to eliminating any structure that does not have a 1.34-Å C–N bond distance; there is no other way to apply this constraint correctly. Similarly, the partial double-bond character of the C–N bond necessitated the elimination of any structure permitting rotation about this bond. In contrast, every X-ray diffraction photograph is compatible with multiple interpretations due to limitations intrinsic to the technology of X-ray diffraction photography. Recall the phase problem: in order to determine molecular structure from an X-ray diffraction photograph, one needs both the amplitude and absolute phase of the X-rays that are diffracted by the molecule. But an X-ray diffraction photograph only reveals the amplitudes and *relative* phases of the waves. Thus, each photograph permits several interpretations, each compatible with different absolute phases of the rays.

This brings us to the second factor that contributes to the confidence scientists could have in applications of constraints to reduce the size of the possibility space: the reliability of (interpretations of) the *constraints themselves*. Note that this is different from how confident scientists could be that they had correctly reduced the size of the possibility space through the consideration of each constraint: this second factor expresses the fact that confidence in the correct application of a constraint can vary *even on the assumption* that the constraint itself is reliable. I will argue that component-level constraints were more reliable than interpretations of X-ray diffraction photographs.

Interpretations of X-ray diffraction photographs of biological macromolecules were relatively unreliable because the photographs tended to be blurry.^13^ The size and complexity of biological macromolecules made them difficult to crystallize, so they often lacked the regular structure characteristic of inorganic crystals, which was necessary for the production of a crisp X-ray diffraction photograph. At the time, then, it was prudent for scientists to approach each interpretation of an X-ray diffraction photograph of such macromolecules with a commensurate degree of skepticism. Even if it appeared to be straightforward and obviously correct, as was the case with Astbury’s interpretation of the 5.1-Å reflection in his photograph of alpha keratin, it was always possible that some other interpretation of the photograph could be found. Indeed, this is what eventually happened: Pauling and Crick independently discovered that two or more alpha helices could be coiled together like strands of a rope. This higher-order structure, dubbed the ‘coiled coil’, was responsible for the spot in the diffraction photograph that seemed to indicate a 5.1-Å height for one turn of the helix (Judson 1996).

In contrast, scientists could have relatively high confidence in component-level constraints. Whereas bond distances and angles were also determined by X-ray diffraction studies, these studies were conducted on small molecules, such as glycine (Albrecht and Corey 1939) and alanine (Levy and Corey 1941). Due to the simplicity of these molecules, X-ray diffraction photographs of them tended to be clearer than photos of complex macromolecules such as keratin. Moreover, their simplicity also left less room for alternative possible interpretations of their X-ray diffraction photos, like the coiled-coil explanation for the 5.1-Å spot in Astbury’s photographs. Finally, many bond distances and angles were further validated by subsequent studies. When Pauling was having difficulty finding a structure that conformed with Astbury’s X-ray diffraction photographs, he considered the possibility that he “was making some unjustified assumption about the structural properties of the molecules” (Pauling 1970, p. 1003), i.e. that one of the bond distances or angles in the polypeptide chain he was working with was wrong. So he and Corey performed X-ray diffraction studies on simple molecules over the next 10 years, the results of which only verified these bond distances and angles. Thus, by 1948, Pauling was convinced that “there was nothing surprising about the dimensions of these molecules,” and that his assumptions about the structural properties of the polypeptide chain from 1937 “were to be accepted as correct” (Pauling 1970, pp. 1003–1004). That is, each additional study on simple molecules lent further confirmation to component-level constraints.

Average informativeness is a product of two factors, and there are differences between how component-level constraints and X-ray diffraction photographs perform on each. I have argued that although both component-level constraints and X-ray diffraction photographs enable the elimination of a considerable proportion of the space of possible structures, scientists were warranted in having much more confidence in the former than in the latter. But I have also conceded that X-ray diffraction photographs enable the elimination of a greater proportion of the possibility space than do component-level constraints. Thus, one might object that since the two factors contributing to informativeness pull in opposite directions for each kind of constraint, it is not obvious that the informativeness of component-level constraints is higher, as I have here argued.

Why think that the better performance of component-level constraints on the second factor outweighs their poorer performance on the first? Mistakes in constraint application are costly, so as long as the numbers of structural candidates eliminated by each kind of constraint are sufficiently alike, the constraints that are most highly confirmed should be considered first. Only if the disparity between the numbers of candidate structures eliminated is very high—say, with one constraint eliminating just a few structures, the other eliminating 90% of them—might it make sense to consider a less highly confirmed constraint before a more highly confirmed one. After all, applying a constraint that eliminates just a few structures would not get one much closer to the correct structure, even if the constraint were guaranteed to be reliable. On the other hand, if a constraint eliminates 90% of the possibility space, one might as well apply it. The payoff of being right is high, and if it turns out one is wrong, one would discover so shortly thereafter, and could easily backtrack and start again. Thus, we generally ought to choose constraints that eliminate structures with certainty, or as close to certainty as possible. And applications of component-level constraints do just this. On balance, then, the components-first heuristic was likely to be more efficient than the photos-first one.

## Does the transformed problem reflect the real problem?

I have characterized the mid-twentieth-century problem of protein structure determination as beginning with a space of possible solutions, which may be narrowed down through the successive consideration of component-level constraints and whole-molecule X-ray diffraction photographs. I argued that the constraint selected for consideration at each stage ought to be the most *informative* of those available: it ought to optimize the balance between how many possibilities are eliminated upon its application and how likely it is that this elimination has been conducted correctly. I showed that, other things being equal, component-level constraints were more informative than X-ray diffraction photographs, so the components-first heuristic is likely to be most cost-effective or efficient. Let us now turn to a question raised in “Introduction” section: to what extent does the problem as I have characterized it here reflect the original problems of protein and DNA structure determination? I will show that although my characterization introduces several idealizations, their effect on heuristic selection is either insignificant, or else it enables the heuristic to be further refined.

First, I have assumed that constraints are individuated in a particular way, each eliminating a unique subset of the space of possible structures. In practice, however, there may be several alternative ways to individuate constraints. One may consider two or more of them together, for instance, by applying multiple component-level constraints at once to more quickly reduce the size of the possibility space. Conversely, one may take a given constraint to say less about structure than I have been assuming here. For example, one might extract only coarse-grained information from an X-ray diffraction photograph, such as a helical structure for keratin, without inferring anything finer-grained, such as the distance between repeating subunits. Let us consider each such alternative individuation of constraints in turn.

The aggregation of multiple component-level constraints strengthens the case for the components-first heuristic being most efficient. Recall that a potential worry about my claim that component-level constraints are more informative than X-ray diffraction photographs was that the greater number of structures eliminated by considering X-ray diffraction photographs than by component-level constraints might be sufficient to outweigh the higher confidence scientists were warranted in having in these constraints. Upon aggregating multiple component-level constraints, the structural candidates remaining for further consideration are those lying in the intersection of the spaces delineated by each of the constraints considered in isolation. Thus, the more such constraints we aggregate, the greater the proportion of the possibility space is eliminated by their consideration, and the more we can alleviate this worry. Moreover, the fact that constraints may be aggregated gives us a different way to make the point that, other things being equal, component-level constraints ought to be considered before X-ray diffraction photographs. Instead of considering the informativeness of *individual* such constraints, we may consider the informativeness of all of them taken together, and compare it to the informativeness of all X-ray diffraction photographs. Doing this introduces another idealization, since in practice, scientists would not always work with one type of constraint independently of the other. But it also enables us to see more decisively why the informativeness of component-level constraints is higher than that of X-ray diffraction photographs.

What about the alternative individuation of constraints according to which we extract smaller bits of information from X-ray diffraction photographs, for instance, coarse-grained information about overall structure and finer-grained information about repeat distance? On such an individuation, it is possible that some of bits of information extracted from X-ray diffraction photographs could be more informative than some component-level constraints. For instance, the helical structure suggested by the X pattern in Astbury’s photographs was relatively highly confirmed: the low resolution of X-ray diffraction photography made it easier to extract such information than finer-grained information about bond lengths and angles. Nevertheless, if the argument in “A (concrete) heuristic: average informativeness of constraints on molecular structure” section is correct, there would be few such instances: most component-level constraints would still be more informative than most (bits of information extracted from) X-ray diffraction photographs.

Second, and related to the previous point, my argument assumes that there is one value of informativeness associated with component-level constraints and another with X-ray diffraction photographs, and I have argued that the former is higher than the latter. We may de-idealize the problem by considering the informativeness of *particular* component-level constraints and (bits of information extracted from) X-ray diffraction photographs.^14^ In so doing, we may produce a more fine-grained heuristic for molecular structure determination. However, and importantly, the coarse grain of the particular heuristic presented here is part of what makes it just that—a general guideline for how to approach the problem, from which one might deviate when there is good reason to do so, rather than a strict set of rules that must be followed without exception.

Third, it is worth emphasizing the *ceteris paribus* clause implicit in a heuristic: *other things being equal*, one ought to consider the most informative constraint at each stage of problem solving. But of course other things tend not to be equal. One assumption I have made is that the scientists selecting between strategies are equally proficient in reasoning with component-level constraints and drawing inferences from X-ray diffraction photographs. In reality, however, scientists who are not sufficiently familiar with reasoning from component-level constraints are likely to make mistakes when applying them; indeed, this is precisely what happened to Bragg, Kendrew, and Perutz. Thus, in some cases, it may well be more efficient to consider X-ray diffraction photographs before component-level constraints.^15^

With this observation in hand, we may use the notion of average informativeness to answer two different questions. First, the one we have been considering: given the state of scientific knowledge and technology at the time, which heuristic should a relatively neutral party—say, a beginning graduate student not yet embedded in a particular methodology—select? But also: which heuristic should a *particular* historical actor, with a specific set of skills and competencies, choose? To answer this second question, in place of the certainty that scientists *in general* were warranted in having in each constraint at the time, we may instead consider the certainty of a *particular historical actor*, ignoring the normative question of whether this certainty was justified given the contemporary state of knowledge. Thus, the heuristic that considers the most informative constraints first can also be applied to individual methodological choices.

## Denouement: explaining Pauling’s and Watson and Crick’s successes

I began this paper by citing a difference in approach to molecular structure determination between Pauling and Watson and Crick on the one hand and Bragg’s group and Franklin on the other: the former adopted a synthetic approach, building structure up from component-level constraints, whereas the latter opted for an analytic one, deriving structure from X-ray diffraction photographs. I then described two alternative heuristics, the components-first and photos-first heuristics, and argued that the former was more efficient than the latter, and thus its use would increase one’s probability of success. Although the primary aim of this paper is normative, I will now turn to a related, descriptive question: can the synthetic and analytic approaches adopted by the historical actors be understood as exemplifying the components-first and photos-first heuristics respectively? And if so, can Pauling’s and Watson and Crick’s successes be, at least in part, attributed to their having adopted the heuristic likeliest to generate the correct structure in the shortest period of time? Although a comprehensive answer to this question would require a depth of historical analysis that is beyond the scope of this paper, I would like to at least gesture toward why I think it is *yes*.

Schindler (2008) has argued convincingly that, contrary to philosophical orthodoxy, Pauling, and later Watson and Crick, largely *ignored* direct empirical evidence—that is, X-ray diffraction photographs—for the structures of protein and DNA respectively. They instead focused almost exclusively on building models based on known bond types, lengths, and angles, ensuring that these models satisfied stereochemical considerations. This model-building approach can be understood as a particular instantiation of the components-first heuristic, which provided a concrete way to prioritize component-level constraints in scientists’ reasoning.^16^ In contrast, Bragg’s group and Franklin were skeptical of the model-building approach, instead prioritizing X-ray diffraction evidence in their search for structure. Schindler shows that both Pauling and Watson and Crick generally took into consideration component-level constraints first, building models on their basis, and comparing them with X-ray diffraction photographs afterwards. In contrast, Bragg’s group and Franklin both began by analyzing X-ray diffraction photographs, checking their results against stereochemical constraints only thereafter. Here I restrict my attention to Schindler’s argument for the DNA case, although the argument for the protein case is similar.

Schindler synthesizes historians’ accounts of the discovery of DNA with historical actors’ autobiographical recollections of their research during this period.^17^ He contrasts Watson and Crick’s model-building strategy with Franklin’s attempt to derive the structure of DNA from her X-ray diffraction photographs (Schindler 2008, pp. 630–33). Schindler cites Crick describing his and Watson’s approach as follows:

What Pauling did show us was that exact and careful model building could embody constraints that the final answer *had in any case to satisfy.* Sometimes this could lead to the correct structure, *using only a minimum of the direct experimental* [X-ray] *evidence* (Crick 1988, p. 60, cited in Schindler 2008, p. 632; Schindler’s emphasis).

According to Crick, then, scientists were so confident in component-level constraints that the correct structure “had” to satisfy these constraints.

Crick also recognized that X-ray diffraction photos could be misleading:

There’s a perfectly sound reason – it isn’t just aesthetics or because we thought it was a nice game—why you should use the *minimum* of experimental evidence. The fact is, you remember, that we knew that Bragg and Kendrew and Perutz had been *misled* by the experimental evidence. And therefore every bit of experimental evidence *we* had got at any one time we were prepared to throw *away*, because we said it may be misleading just the way that 5.1 reflection in alpha keratin was misleading […] The point is that evidence can be unreliable, and therefore you should use as little of it as you can. (Crick, quoted in Judson (1996); original emphasis).

Since the structure had to satisfy component-level constraints, while X-ray diffraction photographs could be misleading, it made sense to begin by constructing a model that satisfied these constraints, consulting diffraction photos only thereafter.

Crick contrasts his and Watson’s approach with how Franklin and Maurice Wilkins, also at King’s College, worked^18^:

The King’s workers were reluctant to be converted to such an approach. Rosalind [Franklin], in particular, wanted to use her experimental data as fully as possible. I think she thought that to guess the structure by trying various models, using a minimum of experimental facts, was too flashy (Crick 1988, p. 60, cited in Schindler 2008, pp. 632–33).

Wilkins concurs:

*Our main mistake was to pay too much attention to experimental evidence*. Nelson won the battle of Copenhagen by putting his blind eye to the telescope so that he did not see the signal to stop fighting. In the same way, scientists sometimes should use the Nelson Principle and *ignore experimental evidence* (Wilkins 2003, p. 166, cited in Schindler 2008, p. 633; Schindler’s emphasis).

I encouraged Bruce Fraser, in our lab, to try out his ideas in a model. Rosalind dismissed our excitement by saying that model-building is what you do *after* you have found the structure (Wilkins 2003, p. 160, cited in Schindler 2008, p. 632; Schindler’s emphasis).

According to PhD student Raymond Gosling, Franklin’s collaborator, Franklin reasoned that the synthetic, model-building approach would be akin to “*speculation*;” in contrast, using their analytic approach, they could “let the spots on this photograph tell [them] what the structure is.” (Gosling, quoted in Judson (1996, p. 127), my emphasis). Thus, whereas Watson and Crick generally applied component-level constraints first, Franklin took them into account only after giving full consideration to her X-ray diffraction photographs. That is, Watson and Crick, but not Franklin, adopted what I have argued was the most cost-effective heuristic for determining the structure of DNA.

If this is right, can Watson and Crick’s adoption of this heuristic explain their success? Developing an understanding of why a historical episode occurred the way it did is difficult. Numerous overlapping and complex factors contribute to the particular ways in which events unfold. These factors cannot always be identified, teased apart, or simplified, and counterfactual reasoning supporting the dominance of one causal explanation over another is necessarily defeasible. With this in mind, we may understand Watson and Crick’s having used the components-first heuristic, while Franklin adopted the photos-first one, to provide the following sort of explanation for their success. Having adopted a heuristic that was more cost-effective than Franklin’s, Watson and Crick were more likely to solve the structure of DNA before her. The fact that they did indeed solve this structure first does not of course *prove* that their use of this heuristic was responsible for their success. Perhaps they made fewer mistakes in their reasoning than did Franklin, maybe social factors such as a strained relationship with Wilkins or being a woman in a male-dominated field significantly disadvantaged Franklin, or perhaps Watson and Crick just got lucky. But given that Watson and Crick’s having adopted this heuristic increased the likelihood of their success, we may consider their adoption of this heuristic, together with other proposals, a defeasible explanation for it. This does not undermine other possible explanations, which are all compatible with this one. For instance, even if a misogynistic climate and a strained relationship with Wilkins imposed structural constraints that severely restricted what Franklin was able to accomplish,^19^ her having adopted a less cost-effective heuristic might have further impeded her progress. Thus, adding this as an additional defeasible explanation enriches our understanding of this significant historical episode.

## Footnotes

1 More precisely, Pauling discovered one way in which a chain of amino acids can fold in three dimensions, what we refer to as the *secondary structure* of protein. The nature of the problem of protein structure that Pauling solved is discussed in “The transformed problem: reducing the space of structural possibilities by applying constraints on structure” section.

2 ‘Bottom-up’ and ‘top-down’ are here used in the information processing sense: as will become clear, I am not drawing on the distinction between theory and data, but between building up a whole from component parts and breaking it down.

3 In this case, whether they were single or double bonds.

4 Pauling also worked on the structure of DNA, but only intermittently. He was more focused on other projects, and the three-stranded model he eventually came up with was a rather hurried effort. We will not consider it here.

5 There is a longstanding debate about *how much* evidence scientists should collect, given a finite amount of time for an investigation, and what the relationship is between this and the ideal limit of what they should do given an infinite amount of time. See Heesen (2015) for a review of the literature and original proposal. The question I address in this paper is different. I assume the availability of a common set of constraints on structure and ask how the order in which different kinds of constraints from that set are consulted affects how quickly the investigation can be concluded.

6 In a similar vein, Craver (2007, Ch. 7) characterizes the process of determining mechanistic explanations in neuroscience as eliminating possible explanations.

7 If bond lengths and angles are allowed to take on any positive real value, the number of possible structures is infinite. The more we restrict permissible values of these variables—for instance, by setting upper and lower bounds on bond lengths and discretizing bond lengths and angles—the fewer the initial number of possibilities. And, in practice, the possibilities would be so restricted. In any case, the number is very large, and that is all that is necessary for the purposes of the argument in this paper.

8 This example is adapted from Pauling’s *Nature of the Chemical Bond* (1960, pp. 281–82).

9 Bond type and length are, of course, related. For instance, single bonds are longer than double bonds. I separate them here because they provide different sorts of constraints on structure.

10 Although Perutz and Kendrew, at Bragg’s lab, also performed diffraction studies of the proteins hemoglobin and myoglobin, Astbury’s photographs were most widely influential, since keratin is a much simpler molecule and thus yielded clearer diffraction images.

11 Average informativeness is inspired by (but not identical to) the notion of informational entropy, a measure for average amount of information, which originated in Claude Shannon’s (1948) mathematical theory of communication.

12 Discussion of this *ceteris paribus* clause follows in “Does the transformed problem reflect the real problem?” section. It will henceforth generally be omitted for the sake for brevity.

13 See, for instance, Astbury’s photos (Figs. 3, 4). In particular, contrast Astbury’s photo of DNA (Fig. 4), which Franklin discovered was of a mixture of the A and B forms, with Franklin’s photos (Fig. 5). It is important to note that while Astbury’s photos were widely available, Franklin’s were published only in the same issue of *Nature* as Watson and Crick’s structure of DNA (Franklin and Gosling 1953).

14 We could further de-idealize by including other constraints on structure that I have not considered here. For instance, we might also include non-bonding interactions as additional constraints on structure, assigning them values of informativeness as outlined in “A (concrete) heuristic: average informativeness of constraints on molecular structure” section.

15 An additional consideration is how much demand on resources is associated with each kind of constraint. Since the process of producing and interpreting X-ray diffraction photographs was notoriously difficult and time-consuming, thus detracting from efficiency, taking this into consideration would strengthen the case for considering component-level constraints first.

16 In fact, I have argued elsewhere that model-building further contributed to the efficiency of the components-first strategy. Because bond lengths and angles were physically instantiated in the pieces from which models were constructed, they were automatically prioritized; scientists using these models could not help but be constrained by them first. Moreover, model-building served as a cognitive aid, helping scientists to more quickly eliminate possible structures (Bolinska 2015).

17 The two most prominent histories of these cases are Judson (1996) and Olby (1974). Other histories have more specific focuses: Hager (1995) on Pauling’s work, Hall (2014) on Astbury, and Maddox (2002) and Sayre (1975) on Franklin. For autobiographical accounts, see Watson (1968, 1980), Crick (1988), Wilkins (2003), and Pauling (1970).

18 Franklin and Wilkins had a notoriously strained relationship. Thus, although they were both at King’s, they did not collaborate closely.

19 This is the explanation proposed by Sayre (1975), but challenged by Judson (1996). Judson interviewed several of Franklin’s contemporary King’s female scientists. They reported that King’s was a good place for women at the time, and rejected the narrative of Franklin as the wronged heroine (1996, pp. 625–27).
